# Development of a Novel Series of Anticancer and Antidiabetic: Spirothiazolidines Analogs

**DOI:** 10.3390/molecules24132511

**Published:** 2019-07-09

**Authors:** Eman M. Flefel, Walaa I. El-Sofany, Reem A.K. Al-Harbi, Mahmoud El-Shahat

**Affiliations:** 1 Department of Photochemistry, Chemical Industries Research Division, National Research Centre, 33 EL-Bohouth St., Dokki 12622, Giza, Egypt; 2 Department of Chemistry, College of Science, Taibah University, Al-Madinah Al-Monawarah 1343, Saudi Arabia

**Keywords:** azaspiro[4.5]decan-3-one, spirothiazolopyridines, MCF-7, HepG-2, alpha-amylase inhibitor, alpha-glucoidase inhibitor

## Abstract

4-(4-Aminophenyl)-1-thia-4-azaspiro[4.5]decan-3-one **1** was prepared and allowed to react with nitrogen nucleophiles to give the corresponding hydrazones **2**–**4**. Further, compound **1** underwent diazotization and afforded the parallel hydrazono derivative **5**; moreover, compound **1** refluxed with active methylene derivatives yielded the corresponding aminospirothiazolo pyridine–carbonitrile derivative **6** and spirothiazolopyridinone–carbonitrile derivative **7**. Condensation of spirothiazolidine **1** with 4-chlorobenzaldehyde gave the corresponding spiro arylidiene derivative **8**, which was utilized as a component of Micheal addition to react with excess of nitrogen nucleophiles to yield novel ring frameworks 4-(3*′*-(4-chlorophenyl)–spiro [cyclohexane-1,5*′*-pyrazolo[3,4-*d*]thiazol]-6*′*(*1′H*)-yl)aniline (**9)** and 4-(3*′*-(4-chlorophenyl)-*6′H*- spiro[cyclohexane-1,5*′*-thiazolo[5,4-*d*]isoxazol]-6*′*-yl)aniline **(10)**. Finally, when spirothiazolo pyridinone–carbonitrile derivative **7** sodium salt generated in situ was reacted with different alkyl halides, it produced the corresponding *N*-derivatives **12**–**16**. Three compounds, **6**, **14**, and **16**, showed high significantly anticancer activities compared with Doxorubicin® (positive control) against human breast carcinoma (MCF-7) and human liver carcinoma (HepG-2) cell lines. On the other hand, compounds **6** and **9** showed higher therapeutic indices for both of alpha-amylase inhibitor and alpha-glucosidase inhibitor than the other tested compounds compared with the antidiabetic Acarbose (positive control).

## 1. Introduction

Cancer is a horrible disease, which concerns the medical community all over the world and is likely to become the principal cause of death in most developed countries by 2030 [1,2,3,4,5,6]. Development of resistance is a key related issue commonly observed in drug therapy [7,8,9,10]. Despite the great efforts in cancer research, resistance is currently insufficient [11,12,13,14,15,16]. Therefore, there is a crucial need to logically design novel, molecularly targeted antineoplastic treatments, which are more selective, preferably less toxic, and eventually more effective than conventional therapies [17].

Diabetes mellitus is a metabolic unrest primarily characterized by high blood glucose level [18]. Diabetes mellitus was initially counted as a disease of slight significance to world health, but now it is considered as an epidemic and one of the major principal threats to human health in the 21st century. The World Health Organization postulates that the Middle East area will possess the highest prevalence rate of diabetes, growing at 163% by the year 2030 [19]. The dramatic increase in the number of diabetic patients is due to changes in lifestyle and behavioral factors, such as sedentary lifestyle, excessive feeding, and obesity. After 1995, a number of new classes of pharmacological agents [20,21,22] were introduced in the market. The classes currently available are insulin and insulin analogues for type-1/type-2 diabetes [23], sulfonylureas [24,25], glinides [22], biguinides [26,27], glitazones (thiazolidine diones) [28], and α–glycosidase inhibitors for type-2 diabetes (T2D) [29]. Scientific researchers have suggested there is a common link between cancer and diabetes that is related to sugar. Research suggests people who have diabetes have a higher risk of developing various forms of cancers [30]. Sugar plays a vital role in how our bodies process nutrients needed for energy and how our cells are fed [31]. Research has found cancer cells thrive on sugar, especially processed sugars like high fructose corn syrup and white sugar [32].

In search of bioactive entities, we found that compounds comprising thiazole moiety which is easily metabolized inside the body are a base structure in many synthetic drugs that have been applied in the therapy of some diseases [33,34,35,36,37,38,39]. The chemistry of heterocyclic candidates incorporating the thiazole nucleus was particularly interesting due to their potential application in medicinal chemistry as antidiabetic [40,41], anti-inflammatory [42,43], antibacterial [42,44,45,46], anticonvulsant [47,48,49], anticancer [50,51,52,53,54], and anti-HIV [55,56,57,58,59]. Otherwise, heterocyclic analogs containing a Spiro-thiazolidines skeleton have a significant place in chemotherapy due to them exhibiting a broad spectrum of potent pharmacological activities [60,61,62,63,64,65].

As a continuation to our program directed towards the synthesis of useful chemotherapeutic agency [66,67,68,69,70,71,72,73,74,75,76,77,78], we decided to explore other spirothiazolidinone derivatives for multiple potential biological activities. The current study was designed to locate novel important scaffold spiro (cyclohexane-thiazolidine) derivatives that can inhibit human cancer targets MCF-7 and HepG-2. Further, we studied the antidiabetic activity intends to screen alpha-amylase and alpha-glucoidase inhibition activity. Hopefully, the newly synthesized spiro derivatives will have the capability to target the disease without harming healthy tissue.

## 2. Results

### 2.1. Chemistry

**Scheme 1:**
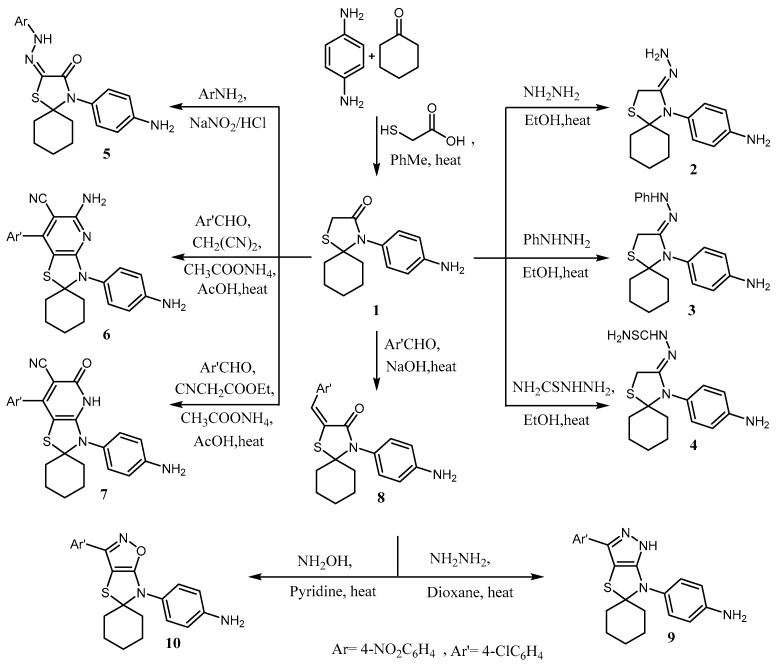
Synthesis of compounds **1**–**10**.

In continuation of our earlier interest on the synthesis of a wide range of applicable heterocyclic compounds [11,12,13,14,15,16,64,65,66,67,68,69,70,71,72,73,74,75,76,77], the starting compound 4-(4-aminophenyl)-1-thia-4-azaspiro [4.5]decan-3-one **(1)** was prepared by condensation between cyclohexanone, p-phenyllenediamine, and thioglycolic acid in dry toluene. Compound **1** was reacted with nitrogen nucleophiles such as hydrazine hydrate, phenylhydrazine, and/or thiosemicarbaxzide to give the hydrazones **2**–**4**, respectively. The IR and ^13^C-NMR spectra of compounds **2**–**4** displayed the absence of the C=O group and the presence of a new signal discriminatory for NH_2_ and NH groups in both IR and ^1^H-NMR spectra, in addition to the presence of the C=S signal in ^13^C-NMR for compound **4** at δ 179.69 ppm (Scheme 1; c.f. experimental). Further, compound **1** underwent diazotization condition in the presence of 4-nitroaniline, which afforded the hydrazono derivative 4-(4-aminophenyl)-2-(2-(4-nitrophenyl) hydrazono)-1-thia-4-azaspiro[4.5]decan-3-one **(5)** in good yield. The ^1^H-NMR spectrum for hydrazono derivative **5** exhibited the absence of two signals specified to active thiazolomethylene protons and the presence of new signals distinguished for the NH group at δ 7.33 ppm (s, 1H; D_2_O exchangeable); (Scheme 1; c.f. experimental). Furthermore, compound **1** was refluxed with active methylene derivatives, namely, malononitrile and/or ethyl cyanoacetate in the presence of both 4-chlorobenzaldehyde and ammonium acetate, which yielded the corresponding aminospirothiazolo pyridine–carbonitrile derivative **6** and spirothiazolo pyridinone–carbonitrile derivative **7**, respectively. The IR spectrum of compound **6** showed absorption bands characteristic for new C≡N and NH_2_ groups at 3122 and 2210 cm^−1^, respectively, in addition to the disappearance of the signal characteristic for the C=O group. The ^1^H-NMR spectrum for derivative **6** showed absence of signals specified to active thiazolomethylene protons in addition to the appearance of new signals for the new NH_2_ group at δ 8.61 ppm, exchangeable with D_2_O. Furturmore, ^13^C-NMR for compound **6** showed a specified signal for the C≡N group at δ 114.69 ppm with the absence of the signal for the C=O group (Scheme 1; c.f. experimental).

The structure of derivative **7** was confirmed on the basis of its spectral data. The IR spectrum for **7** showed the absorption band characteristic for (C=O; amide), (C≡N), and (NH; amide) at 1658, 2214, and 3119 cm^−1^, respectively. The ^1^H-NMR of compound **7** revealed a new D_2_O exchangeable signal at δ 7.01 due to the NH group (Scheme 1; c.f. experimental).

Condensation of spirothiazolidine **1** with 4-chlorobenzaldehyde gave the corresponding arylidiene derivative **8** in quantitative yields. Arylidiene derivative **8** was confirmed by the presence of a new singlet signal at δ 8.01 ppm in the ^1^H-NMR spectrum corresponding to excocyclic vinylic proton (Scheme 1; c.f. experimental). The arylidiene of spiro thiazolidine **8** contained the *α*,*β*-unsaturated function system which has been used as a component of Micheal addition to react with excess of nitrogen nucleophiles, namely, hydrazine hydrate and/or hydroxyl amine hydrochloride to yield novel ring frameworks 4-(3*′*-(4-chlorophenyl)-spiro[cyclohexane-1,5*′*- pyrazolo[3,4-*d*]thiazol]-6*′* (*1′H*)-yl)aniline **9** and 4-(3*′*-(4-chlorophenyl)-*6′H*-spiro[cyclohexane-1, 5′-thiazolo [5,4-*d*]isoxazol]-6*′*-yl)aniline **10** (Scheme 1; c.f. experimental). The ^1^H-NMR spectra for compounds **9** and **10** were devoid of the excocyclic vinylic proton signal, which was present in its precursor, a confirmed heterocyclization reaction. Additionally, IR and ^13^C-NMR spectra free from the C=O group signal and agree with the proposed structures (Scheme 1; c.f. experimental).

**Scheme 2:**
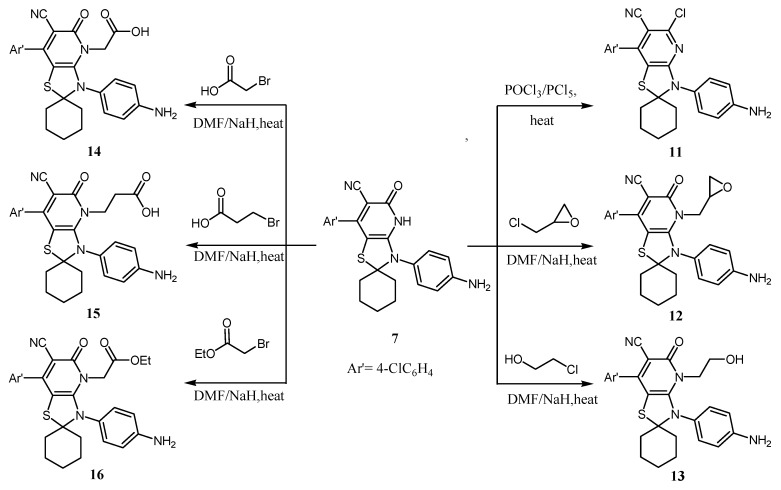
Synthesis of compounds **11**–**16.**

On the other hand, the spirothiazolopyridinone–carbonitrile derivative **7** was utilized as a key starting material for the synthesis of novel heterocyclic ring systems. The pyridinone derivative **7** was reacted with a mixture of PCl_5_/POCl_3_ on water bath afforded the corresponding chloro derivative **11**. Compound **11** was elucidated by the absence of the (C=O) group in both the IR and ^13^C-NMR spectra. In addition to the absence of the NH signal in both the IR and ^1^H-NMR spectra, its mass spectrum afforded the molecular ion peak M^+^ at *m/z* 466 as well as the presence of an isotopic pattern of 2 chlorine atoms in agreement with its molecular formula (Scheme 2; c.f. experimental).

Finally, spirothiazolopyridinone–carbonitrile derivative **7** sodium salt generated in situ was reacted with epichlorohydrine, chloroethanol, bromoacetic acid, bromopropionic acid, and/or ethyl bromoacetate and produced the corresponding *N*-derivatives, **12**–**16**, respectively (Scheme 2; c.f. experimental). The ^13^C-NMR and IR spectra for the abovementioned *N*-derivatives **12**–**16** detected the attack position which was on the nitrogen and not oxygen atom. Further, NMR spectra exhibited signals for oxiran-2-ylmethyl, hydroxyethyl, acetic acid, propionic acid, and ethyl acetate groups (Scheme 2; c.f. experimental).

### 2.2. Anticancer

**Figure 1:**
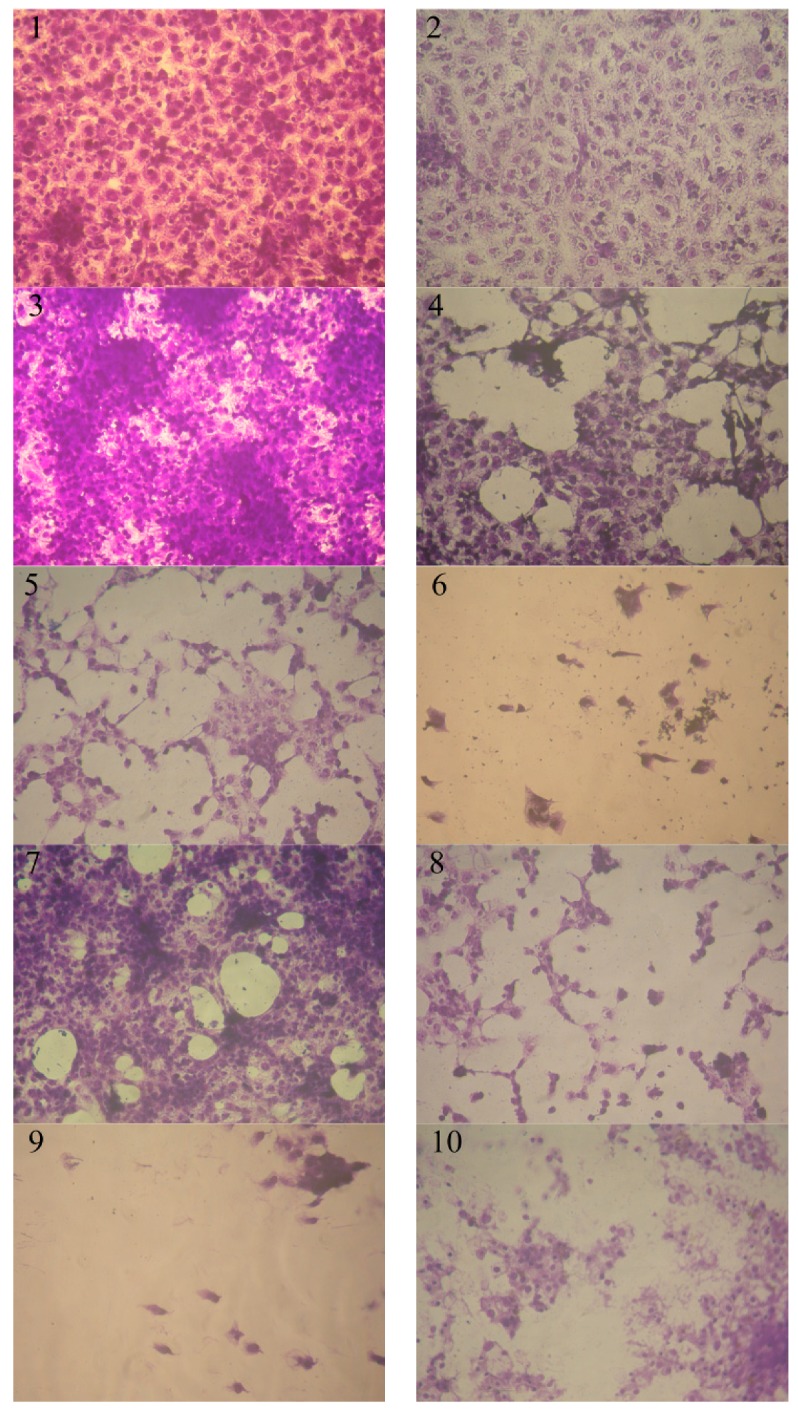
Effect of various concentrations of tested compounds on HepG-2 cell inhibition activity. (1) Control HepG-2 (2) compound **6** (7.8 µg/mL), (3) compound **14** (7.8 µg/mL), (4) compound **14** (125 µg/mL), (5) compound **6** (125 µg/mL), (6) compound **6** (500 µg/mL), (7) compound **12** (7.8 µg/mL), (8) compound **12** ( 125 µg/mL), (9) compound **16** (500 µg/mL), and (10) compound **14** (500 µg/mL).

**Figure 2:**
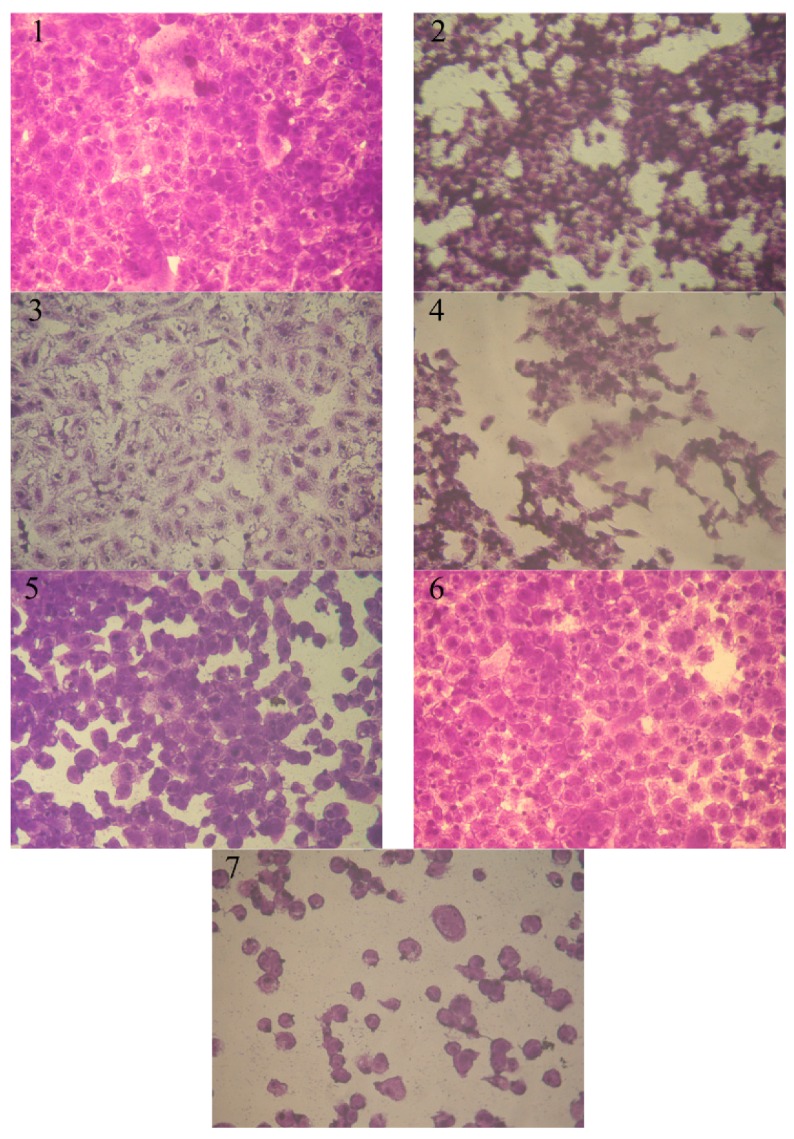
Effect of various concentrations of tested compounds on MCF-7 cell Inhibition activity. (1) Control MCF-7 (2) compound **14** (500 µg/mL), (3) compound **6** (7.8 µg/mL), (4) compound **6** (500 µg/mL), (5) compound **12** (7.8 µg/mL), (6) compound **12** (31.25 µg/mL), and (7) compound **12** (500 µg/mL).

Anticancer bioassays were carried out to test compounds **4**, **6**, **8**, **12**, **14**, and **16** towards the MCF-7 and HepG-2 cell lines. The HepG-2 and MCF-7 cell lines’ inhibition activities are given in Figure 1 and Figure 2. The concentration required for 50% inhibition of cell viability (IC_50_) was calculated from the graph shown the relation between concentrations of tested compound (µg/mL) and cell viability %, and the results are given in Table 1 (for details see Supplementary Materials). It was obvious that at different concentrations (3.90, 7.8, 15.6, 31.25, 62.5, 125, 250, and 500 µg/mL), compounds **6**, **14**, and **16** showed higher therapeutic indices for both breast cell line MCF-7 and liver cell line HepG-2 than the other tested compounds, and the results were compared with the anticancer drug Doxorubicin® (positive control).

### 2.3. Antidiabetic

Antidiabetic bioassays were carried out to test compounds **1**, **2**, **6**, **7**, **8**, **9, 13**, and **16** as alpha-amylase inhibitor and alpha-glucosidase inhibitor. The concentrations of the tested compounds, which exhibited 50% of their activities at the same condition (IC_50_), were determined, and the results are given in Table 2. It was obvious that by comparison with the antidiabetic Acarbose (positive control) at different concentrations (7.81, 15.63, 31.25, 62.5, 125, 250, 500, and 1000 µg/ml), compounds **6** and **9** showed a higher therapeutic effect for both alpha–amylase inhibitor and alpha–glucosidase inhibitor than the other tested compounds. Further, both compounds **6** and **9** showed higher inhibition against alpha-glucosidase than alpha-amylase.

## 3. Materials and Methods

### 3.1. General Information

Melting points were measured using an Electro-Thermal IA 9100 digital melting point apparatus (Büchi, Flawil, Switzerland) and are uncorrected. Infrared spectra were recorded on a Perkin-Elmer 1600 FTIR (Perkin-Elmer, Waltham, MA, USA) discs. NMR spectra were determined on a Jeol-Ex-500 NMR spectrometer (JEOL, Tokyo, Japan), and chemical shifts were expressed as part per million; (δ values, ppm) against TMS as internal reference, National Research Center, Cairo, Egypt. The mass spectra were run at 70 eV with a Finnigan SSQ 7000 spectrometer (Thermo Electron Corporation, Madison, WI, USA) using EI, and the values of m/z are indicated in Dalton. Elemental analyses were performed on a Perkin-Elmer 2400 analyzer (Perkin-Elmer) and were found within the accepted range (±0.30) of the calculated values. Reaction monitoring and verification of the purity of the compounds was done by TLC on silica gel precoated aluminum sheets (type 60 F254, Merck, Darmstadt, Germany). All solvents and chemical reagents were purchased from Aldrich (Munich, Germany).

### 3.2. Chemistry

#### 3.2.1. 4-(4-aminophenyl)-1-thia-4-azaspiro[4.5]decan-3-one (**1**)

A mixture of cyclohexanone (0.98 mL, 0.01 mol), *p*-phenyllenediamine (0.01 mol), and thioglycolic acid (0.92 mL, 0.01 mol) in dry toluene (50 mL) was refluxed for 10 h. The solution was concentrated and the formed solid was filtered off, dried, and crystallized from dioxane/methanol to give compound **1**. Pale yellow powder, Yield 92%; m.p.170–172 °C; IR (KBr, υ, cm^−1^): 3230 (NH_2_), 1666 (C=O); ^1^H-NMR (DMSO): δ (ppm) 1.52–2.00 (m, 10H, 5CH_2_), 3.41 (d, *J* = 8.06 Hz, 1H, CH_2_), 3.48 (d, *J* = 8.05 Hz, 1H, CH_2_), 3.87 (s, 2H, NH_2_; D_2_O exchangeable), 7.11–7.67 (m, 4H, Ar-H); ^13^C-NMR spectrum (CDCl_3_, δ ppm): 22.41, 25.64, 35.28, 35.61, 80.49, 117.08, 126.95, 131.38, 140.04, 169.98; MS, *m/z* (%): 262 (M^+^, 100); Analysis calc. for C_14_H_18_N_2_OS (262.37):C, 64.09; H, 6.92; N, 10.68; S, 12.22. Found: C, 63.84; H, 6.66; N, 10.42; S, 11.97.

#### 3.2.2. General procedure for the synthesis of compounds **2** and **3**

A mixture of compound **1** (0.01 mol) and hydrazine hydrate or phenylhydrazine (0.02 mol) was refluxed in absolute ethanol (30 mL) for 6 h. The obtained solid was filtered off, dried, and crystallized from the proper solvent to give compounds **2** and **3**.

##### 4-(3-hydrazono-1-thia-4-azaspiro[4.5]decan-4-yl)aniline (**2**)

From dioxane. Pale yellow fine crystals, Yield 83%; m.p. 263–265 °C; IR (KBr, υ, cm^−1^): 3343, 3232 (2NH_2_); ^1^H-NMR (DMSO): δ (ppm) 1.54–1.90 (m, 10H, 5CH_2_), 3.57 (d, *J* = 6.60 Hz, 1H, CH_2_), 3.58 (d, *J* = 6.61 Hz, 1H, CH_2_), 4.24 (s, 2H, NH_2_; D_2_O exchangeable), 5.24 (s, 2H, NH_2_; D_2_O exchangeable), 6.55–6.77 (m, 4H, Ar-H); ^13^C-NMR spectrum (DMSO, δ ppm): 22.46, 22.71, 31.96, 35.28, 78.82, 116.09, 125.43, 134.93, 142.48, 151.30; MS, *m/z* (%): 276 (M^+^, 34); Analysis calc. for C_14_H_20_N_4_S (276.14): C, 60.84; H, 7.29; N, 20.27; S, 11.60. Found: C, 60.58; H, 7.02; N, 20.02; S, 11.36.

##### 4-(3-(2-phenylhydrazono)-1-thia-4-azaspiro[4.5]decan-4-yl)aniline (**3**)

From methanol. Brown needle fine crystals, Yield 79%; m.p. 280–282 °C; IR (KBr, υ, cm^−1^): 3342 (NH_2_), 3226 (NH); ^1^H-NMR (DMSO): δ (ppm) 1.53‒1.91 (m, 10H, 5CH_2_), 3.58 (d, *J* = 6.62 Hz, 1H, CH_2_), 3.63 (d, *J* = 6.61 Hz, 1H, CH_2_), 5.28 (s, 2H, NH_2_; D_2_O exchangeable), 6.27(s, 1H, NH; D_2_O exchangeable), 6.57‒6.78(m, 4H, Ar-H), 7.28‒7.59 (m, 5H, Ar-H); ^13^C-NMR spectrum (DMSO, δ ppm): 22.42, 25.67, 33.66, 35.24, 78.78, 115.19, 116.05, 124.00, 125.39, 128.82, 134.89, 142.44, 143.59, 151.33; MS, *m/z* (%): 352 (M^+^, 47). Analysis calc. for C_20_H_24_N_4_S (352.17): C, 68.15; H, 6.86; N, 15.89; S, 9.10. Found: C, 67.89; H, 6.61; N, 15.63; S, 8.82.

#### 3.2.3. 2-(4-(4-aminophenyl)-1-thia-4-azaspiro[4.5]decan-3-ylidene)hydrazine-1-carbothioamide (**4**)

Two milliliters of concentrated HCl was added to a solution of compound **1** (2.6 g, 0.01 mol), thiosemicarbazide (0.01 mol) in absolute ethanol (30 mL). The reaction mixture was refluxed for 3 h, then left to cool and the formed solid was filtered off, washed with water, and crystallized from dioxane to give compound **4**. Pale yellow needle fine crystals, Yield 67%; m.p. 255–257 °C; IR (KBr, υ, cm^−1^): 3332, 3226 (2NH_2_), 3121 (NH); ^1^H-NMR (DMSO): δ (ppm) 1.54‒1.90 (m, 10H, 5CH_2_), 3.59 (d, *J* = 6.61 Hz, 1H, CH_2_), 3.62 (d, *J* = 6.61 Hz, 1H, CH_2_), 5.27(s, 2H, NH_2_; D_2_O exchangeable), 6.64‒6.86 (m, 4H, Ar-H), 7.11 (s, 2H, NH_2_; D_2_O exchangeable), 8.21 (s, 1H, NH; D_2_O exchangeable); ^13^C-NMR spectrum (DMSO, δ ppm): 22.48, 25.73, 33.71, 35.29, 78.81, 116.11, 125.44, 134.95, 142.49, 152.85, 179.69; MS, *m/z* (%): 335 (M^+^, 22). Analysis calc. for C_15_H_21_N_5_S_2_ (335.12): C, 53.70; H, 6.31; N, 20.88; S, 19.11. Found: C, 53.44; H, 6.07; N, 20.63; S, 18.85.

#### 3.2.4. 4-(4-aminophenyl)-2-(2-(4-nitrophenyl)hydrazono)-1-thia-4-azaspiro[4.5]decan-3-one (**5**)

A solution of hydrochloric acid (6 mL); 4-nitroaniline (0.01 mol) and an aqueous solution (3 mL) of sodium nitrite (0.72 g, 0.015 mol) was stirred at 0 °C for 1 h, followed by addition of (0.01 mol) of compound **1** in 10 mL pyridine, and stirring was continued at 0 °C for 2 h. The resulting product was filtered off, washed with water, dried, and crystallized from dioxane to give compound **5**. White powder, m.p. over 300 °C.; IR (KBr, υ, cm^−1^): 3211 (NH_2_), 3111 (NH), 1651 (C=O); ^1^H-NMR (DMSO): δ (ppm) 1.52‒1.92 (m, 10H, 5CH_2_), 5.30 (s, 2H, NH_2_; D_2_O exchangeable), 6.56‒6.81 (m, 4H, Ar-H), 7.33 (s, 1H, NH; D_2_O exchangeable),7.47‒7.79 (m, 4H, Ar-H); ^13^C-NMR spectrum (DMSO, δ ppm): 22.41, 25.65, 34.68, 82.18, 115.95, 116.45, 126.33, 126.94, 128.12, 129.96, 143.64, 143.82, 150.33, 172.58; MS, *m/z* (%): 411 (M^+^, 28). Analysis calc. for C_20_H_21_N_5_O_3_S (411.14): C, 58.38; H, 5.14; N, 17.02; S, 7.79. Found: C, 58.14; H, 4.90; N, 16.78; S, 7.54.

#### 3.2.5. General procedure for the synthesis of compounds **6** and **7**

A mixture of compound **1** (2.6 g, 0.01 mol), 4-chlorobenzaldehyde (1.4 g, 0.01 mol), ammonium acetate (0.02 mol), and malononitrile (0.06 g, 0.01 mol) or ethylcyanoacetate (1.1 g, 0.01 mol) in glacial acetic acid (40 mL) was refluxed for 24 h. The reaction mixture was cooled and poured into water. The formed solid was filtered off, dried, and crystallized from proper solvent to give compounds **6** and **7**.

##### 5*′*-amino-3*′*-(4-aminophenyl)-7*′*-(4-chlorophenyl)-3*′*H-spiro[cyclohexane-1,2*′*-thiazolo[4,5-b] pyridine]-6*′*-carbonitrile (**6**)

From methanol. Brown powder, Yield 81%; m.p. 233–235 °C; IR (KBr, υ, cm^−1^): 3295, 3122 (2NH_2_), 2210 (C≡N); ^1^H-NMR (DMSO): δ (ppm) 1.54‒1.93 (m, 10H, 5CH_2_), 5.31 (s, 2H, NH_2_; D_2_O exchangeable), 6.57‒6.83 (m, 4H, Ar-H), 7.11‒7.37 (m, 4H, Ar-H), 8.61 (s, 2H, NH_2_; D_2_O exchangeable); ^13^C-NMR spectrum (DMSO, δ ppm): 22.40, 25.63, 34.28, 81.48, 91.25, 114.69, 116.45, 124.49, 128.63, 129.57, 129.81, 133.24, 133.92, 135.16, 145.64, 154.02, 154.53, 159.58; MS, *m/z* (%): 477 (M^+^, 67), 479 (M^+^ + 2, 21). Analysis calc. for C_24_H_22_ClN_5_S (447.13): C, 64.35; H, 4.95; Cl, 7.91; N, 15.63; S, 7.16. Found: C, 64.08; H, 4.71; Cl, 7.66; N, 15.38; S, 6.87.

##### 3*′*-(4-aminophenyl)-7*′*-(4-chlorophenyl)-5*′*-oxo-4*′*,5*′*-dihydro-3*′*H-spiro[cyclohexane-1,2*′*- thiazolo[4,5-b]pyridine]-6*′*-carbonitrile (**7**)

From dioxane. Deep yellow powder, Yield 77%; m.p. 225–227 °C; IR (KBr, υ, cm^−1^): 3294 (NH_2_), 3119 (NH), 2214 (C≡N), 1658 (C=O); ^1^H-NMR (DMSO): δ (ppm) 1.53‒1.91 (m, 10H, 5CH_2_), 5.29 (s, 2H, NH_2_; D_2_O exchangeable), 6.61‒6.84 (m, 4H, Ar-H), 7.01 (s, 1H, NH_2_; D_2_O exchangeable), 7.14‒7.40 (m, 4H, Ar-H); ^13^C-NMR spectrum (DMSO, δ ppm): 22.41, 25.62, 34.33, 79.01, 101.16, 114.45, 116.67, 125.09, 129.13, 130.45, 132.31, 136.04, 136.52, 136.66, 143.99, 147.29, 149.75, 162.47; MS, *m/z* (%): 448 (M^+^, 57), 450 (M^+^ + 2, 18). Analysis calc. for C_24_H_21_ClN_4_OS (448.11): C, 64.21; H, 4.71; Cl, 7.90; N, 12.48; S, 7.14. Found: C, 63.93; H, 4.42; Cl, 7.66; N, 12.18; S, 6.87.

#### 3.2.6. 4-(4-aminophenyl)-2-(4-chlorobenzylidene)-1-thia-4-azaspiro[4.5]decan-3-one (**8**)

A mixture of compound **1** (0.01 mol) and 4-chlorobenzaldehyde (0.01 mol) was refluxed in ethanolic NaOH for 2 h. The product was poured onto ice and neutralized with dilute HCl, and then the formed solid was filtered off and crystallized from dioxane to give **8**. Pink powder, Yield 66%; m.p. 293–295 °C; IR (KBr, υ, cm^−1^): 3226 (NH_2_), 1670 (C=O); ^1^H-NMR (DMSO): δ (ppm) 1.52‒1.91 (m, 10H, 5CH_2_), 5.28 (s, 2H, NH_2_; D_2_O exchangeable), 6.62‒6.86 (m, 4H, Ar-H), 7.12‒7.39 (m, 4H, Ar-H), 8.01 (s, 1H, exocyclic vinylic-H); ^13^C-NMR spectrum (DMSO, δ ppm): 22.43, 25.62, 34.43, 82.67, 116.47, 126.93, 126.96, 128.93, 129.97, 130.85, 131.77, 133.74, 137.53, 143.67, 165.61; MS, *m/z* (%): 384 (M^+^, 53), 386 (M^+^ + 2, 16). Analysis calc. for C_21_H_21_ClN_2_OS (384.11): C, 65.53; H, 5.50; Cl, 9.21; N, 7.28; S, 8.33. Found: C, 65.24; H, 5.22; Cl, 8.93; N, 6.99; S, 8.03.

#### 3.2.7. 4-(3*′*-(4-chlorophenyl)-spiro[cyclohexane-1,5*′*-pyrazolo[3,4-d]thiazol]-6*′* (1′H)-yl)aniline (**9**)

A mixture of compound **8** (0.01 mol) and hydrazine hydrate (0.02 mol) was refluxed in 30 mL dioxane for 10 h. The reaction mixture was concentrated under reduced pressure; the formed solid was filtered off, dried, and crystallized from dioxane to give compound **9**. White powder, Yield 69%; m.p. 278–280 °C; IR (KBr, υ, cm^−1^): 3231 (NH_2_), 3131 (NH); ^1^H-NMR (DMSO): δ (ppm) 1.51‒1.92 (m, 10H, 5CH_2_), 5.33 (s, 2H, NH_2_; D_2_O exchangeable), 6.61‒6.87 (m, 4H, Ar-H), 7.35 (s, 1H, NH; D_2_O exchangeable), 7.53‒7.64 (m, 4H, Ar-H); ^13^C-NMR spectrum (DMSO, δ ppm): 22.42, 25.63, 34.38, 78.97, 116.87, 118.48, 125.42, 128.30, 129.46, 129.95, 132.78, 136.37, 139.38, 147.39, 152.60; MS, *m/z* (%): 396 (M^+^, 36), 398 (M^+^ + 2, 11). Analysis calc. for C_21_H_21_ClN_4_S (396.12): C, 63.54; H, 5.33; Cl, 8.93; N, 14.12; S, 8.08. Found: C, 63.26; H, 5.04; Cl, 8.67; N, 13.84; S, 7.81.

#### 3.2.8. 4-(3*′*-(4-chlorophenyl)-6*′*H-spiro[cyclohexane-1,5*′*-thiazolo[5,4-d]isoxazol]-6*′*-yl)aniline (**10**)

A mixture of compound **8** (0.01 mol) and hydroxyl amine hydrochloride (0.5 g, 0.01 mol) was refluxed in pyridine (20 mL) for 10 h. The reaction mixture was cooled, poured into 100 mL water, and neutralized with dilute HCl. The product was filtered off, dried, and crystallized to give compound **10**. Brown powder, Yield 57%; m.p. 290–292 °C; IR (KBr, υ, cm^−1^): 3222 (NH_2_); ^1^H-NMR (DMSO): δ (ppm) 1.51‒1.92 (m, 10H, 5CH_2_), 5.32 (s, 2H, NH_2_; D_2_O exchangeable), 6.60‒6.86 (m, 4H, Ar-H), 7.51‒7.62 (m, 4H, Ar-H); ^13^C-NMR spectrum (DMSO, δ ppm): 22.41, 25.62, 34.41, 81.06, 107.55, 117.67, 125.61, 127.39, 127.91, 129.49, 132.18, 133.47, 145.49, 156.64; MS, *m/z* (%): 397 (M^+^, 44), 399 (M^+^ + 2, 14). Analysis calc. for C_21_H_20_ClN_3_S (397.10): C, 63.39; H, 5.07; Cl, 8.91; N, 10.56; S, 8.06. Found: C, 63.10; H, 4.78; Cl, 8.67; N, 10.27; S, 7.78.

#### 3.2.9. 3*′*-(4-aminophenyl)-5*′*-chloro-7*′*-(4-chlorophenyl)-3*′*H-spiro[cyclohexane-1,2*′*-thiazolo[4,5-b] pyridine]-6*′*-carbonitrile (**11**)

A suspension of compound **7** (0.01 mol), POCl_3_ (3 mL), and PCl_5_ (0.5 gm) was heated in a steam bath for 2 h. The reaction mixture was poured gradually onto crushed ice. The separated solid was filtered off and recrystallized from dioxane to give compound **11**. Black oil. Yield 52%; IR (KBr, υ,cm^−1^): 3219 (NH_2_), 2214 (C≡N); ^1^H-NMR (DMSO): δ (ppm) 1.50‒1.91 (m, 10H, 5CH_2_), 5.30 (s, 2H, NH_2_; D_2_O exchangeable), 6.59‒6.84 (m, 4H, Ar-H), 7.49‒7.60 (m, 4H, Ar-H); ^13^C-NMR spectrum (DMSO, δ ppm): 22.42, 25.64, 34.19, 87.42, 96.01, 115.91, 116.42, 124.34, 128.27, 129.57, 133.72, 139.69, 135.21, 135.58, 141.79, 146.67, 153.26, 156.62; MS, *m/z* (%): 466 (M^+^, 44), 468 (M^+^ + 2, 29), 470 (M^+^ + 4, 4). Analysis calc. for C_24_H_20_Cl_2_N_4_S (466.08): C, 61.67; H, 4.31; Cl, 15.17; N, 11.99; S, 6.86. Found: C, 61.39; H, 4.03; Cl, 14.88; N, 11.69; S, 6.58.

#### 3.2.10. General procedure for the synthesis of compounds **12**‒**16**

To a solution of compound **7** (0.01 mol) in dry DMF (20 mL), sodium hydride (0.24g, 0.01 mol) was added, then the reaction mixture was stirred at 70 °C for 3 h; the reaction mixture was cooled and then epichlorohydrine, chloroethanol, bromoacetic acid, bromopropionic acid, and/or ethyl bromo acetate (0.01 mol) was added, and stirring at room temperature was continued for 5 h. The reaction mixture was evaporated under reduced pressure; the residue was washed with distilled water, filtered off, dried, and recrystallized from dioxane to give compounds **12**‒**16.**

##### 3*′*-(4-aminophenyl)-7*′*-(4-chlorophenyl)-4*′*-(oxiran-2-ylmethyl)-5*′*-oxo-4′,5*′*-dihydro-3*′*H-spiro[cyclo hexane-1,2*′*-thiazolo[4,5-b]pyridine]-6*′*-carbonitrile (**12**)

Deep brown powder, Yield 76%; m.p. 240–242 °C; IR (KBr, υ, cm^−1^): 3228 (NH_2_), 2217(C≡N), 1673 (C=O); ^1^H-NMR (DMSO): δ (ppm) 1.52‒1.92 (m, 10H, 5CH_2_), 2.94 (dd, 1H, *J* = 1.49 Hz; *J* = 4.51 Hz, OCH_2_), 2.98 (dd, 1H, *J* = 4.21 Hz; *J* =4.54 Hz, OCH_2_), 4.30–4.42 (m, 1H, OCH), 4.79 (dd, 1H, *J* = 2.18 Hz; *J* = 5.22 Hz, NCH_2_), 4.82 (dd, 1H, *J* = 2.26 Hz; *J* = 4.98 Hz, NCH_2_), 5.28 (s, 2H, NH_2_; D_2_O exchangeable), 6.60‒6.83 (m, 4H, Ar-H), 7.47‒7.59 (m, 4H, Ar-H); ^13^C-NMR spectrum (DMSO, δ ppm): 22.41, 25.63, 34.33, 45.73, 46.60, 47.94, 79.21, 107.63, 114.57, 116.07, 124.69, 126.68, 129.30, 130.24, 132.92, 135.97, 136.25, 143.18, 143.90, 147.44, 158.22. MS, *m/z* (%): 447 (M^+^- 57, 100), 449 ([M^+^ + 2]-57, 32). Analysis calc. for C_27_H_25_ClN_4_O_2_S (504.14): C, 64.21; H, 4.99; Cl, 7.02; N, 11.09; S, 6.35. Found: C, 63.93; H, 4.72; Cl, 6.74; N, 10.82; S, 6.06.

##### 3*′*-(4-aminophenyl)-7*′*-(4-chlorophenyl)-4*′*-(2-hydroxyethyl)-5*′*-oxo-4*′*,5*′*-dihydro-3′H-spiro[cyclo hexane-1,2*′*-thiazolo[4,5-b]pyridine]-6*′*-carbonitrile (**13**)

Pale yellow powder, Yield 86%; m.p. 208–210 °C; IR (KBr, υ, cm^−1^): 3400 (OH), 3294 (NH_2_), 2211 (C≡N), 1678 (C=O); ^1^H-NMR (DMSO): δ (ppm) 1.50‒1.89 (m, 10H, 5CH_2_), 3.79 (t, *J* = 4.51 Hz, 2H, NCH_2_), 4.58 (t, *J* = 4.53 Hz, 2H, CH_2_O), 5.28 (s, 2H, NH_2_; D_2_O exchangeable), 5.54 (bs, 1H, OH; D_2_O exchangeable), 6.61‒6.84 (m, 4H, Ar-H), 7.46‒7.58 (m, 4H, Ar-H); ^13^C-NMR spectrum (DMSO, δ ppm): 22.42, 25.62, 34.41, 46.76, 59.29, 79.49, 107.72, 114.89, 116.67, 124.72, 126.77, 129.34, 130.28, 135.99, 136.55, 142.48, 143.19, 147.38, 157.87. MS, *m/z* (%): 447 (M^+^- 45, 100), 449 ([M^+^ + 2]-45, 31). Analysis calc. for C_26_H_25_ClN_4_O_2_S (492.14): C, 63.34; H, 5.11; Cl, 7.19; N, 11.36; S, 6.50. Found: C, 63.05; H, 4.85; Cl, 6.91; N, 11.07; S, 6.21.

##### 2-(3*′*-(4-aminophenyl)-7*′*-(4-chlorophenyl)-6*′*-cyano-5*′*-oxo-3*′*,5*′*-dihydro-4′H-spiro[cyclohexane -1, 2*′*-thiazolo[4,5-b]pyridin]-4*′*-yl)acetic acid (**14**)

Brown powder, Yield 69%; m.p. 280–282 °C; IR (KBr, υ, cm^−1^): 3410 (OH), 3288 (NH_2_), 2216 (C≡N), 1740 (C=O), 1672 (C=O); ^1^H-NMR (DMSO): δ (ppm) 1.51‒1.90 (m, 10H, 5CH_2_), 4.87 (s, 2H, NCH_2_), 5.29 (s, 2H, NH_2_; D_2_O exchangeable), 6.65‒6.89 (m, 4H, Ar-H), 7.51‒7.64 (m, 4H, Ar-H), 10.45 (bs, 1H, OH; D_2_O exchangeable); ^13^C-NMR spectrum (DMSO, δ ppm): 22.41, 25.63, 34.43, 43.16, 79.87, 104.69, 114.56, 116.77, 124.69, 126.68, 129.30, 130.33, 132.92, 135.97, 136.25, 142.28, 143.23, 147.49, 161.38, 172.79. MS, *m/z* (%): 447 (M^+^-59, 100), 449 ([M^+^ + 2]-59, 31). Analysis calc. for C_26_H_23_ClN_4_O_3_S (506.12): C, 61.59; H, 4.57; Cl, 6.99; N, 11.05; O, 9.47; S, 6.32. Found: C, 61.31; H, 4.28; Cl, 6.72; N, 10.76; S, 5.93.

##### 3-(3*′*-(4-aminophenyl)-7*′*-(4-chlorophenyl)-6*′*-cyano-5*′*-oxo-3*′*,5*′*-dihydro-4*′*H-spiro[cyclo hexane-1,2*′*-thiazolo[4,5-b]pyridin]-4*′*-yl)propanoic acid (**15**)

Brown powder, Yield 72%; m.p. 200–202 °C; IR (KBr, υ, cm^−1^): 3411 (OH), 3261 (NH_2_), 2214 (C≡N), 1733 (C=O), 1668 (C=O); ^1^H-NMR (DMSO): δ (ppm) 1.50‒1.91 (m, 10H, 5CH_2_), 3. 51 (t, *J* = 4.47 Hz, 2H, CH_2_CO), 4.35 (t, *J* = 4.49 Hz, 2H, NCH_2_), 5.28 (s, 2H, NH_2_; D_2_O exchangeable), 6.66‒6.90 (m, 4H, Ar-H), 7.53‒7.65 (m, 4H, Ar-H), 10.67 (bs, 1H, OH; D_2_O exchangeable); ^13^C-NMR spectrum (DMSO, δ ppm): 22.40, 25.64, 34.36, 35.60, 44.43, 79.93, 107.72, 114.81, 116.70, 124.69, 126.65, 129.32, 130.41, 132.32, 135.97, 136.27, 142.21, 143.11, 147.51, 159.98, 173.47. MS, *m/z* (%): 447 (M^+^-73, 100), 449 ([M^+^ + 2]-73, 30). Analysis calc. for C_27_H_25_ClN_4_O_3_S (520.13): C, 62.24; H, 4.84; Cl, 6.80; N, 10.75; S, 6.15. Found: C, 61.98; H, 4.56; Cl, 6.53; N, 10.46; S, 5.87.

##### Ethyl 2-(3*′*-(4-aminophenyl)-7*′*-(4-chlorophenyl)-6*′*-cyano-5′-oxo-3*′*,5*′*-dihydro-4*′*H-spiro [cyclohexane-1,2*′*-thiazolo[4,5-b]pyridin]-4*′*-yl)acetate (**16**)

Brown powder, Yield 91%; m.p. 190–192 °C; IR (KBr, υ, cm^−1^): 3257 (NH_2_), 2217 (C≡N), 1718 (C=O), 1663 (C=O); ^1^H-NMR (DMSO): δ (ppm) 1.17 (t, *J* = 7.94 Hz, 3H, CH_3_CH_2_O), 1.51‒1.92 (m, 10H, 5CH_2_), 4.17 (q, *J* = 7.96 Hz, 2H, CH_3_CH_2_O), 5.11 (s, 2H, NCH_2_), 5.30 (s, 2H, NH_2_; D_2_O exchangeable), 6.67‒6.92 (m, 4H, Ar-H), 7.54‒7.67 (m, 4H, Ar-H), 10.67 (bs, 1H, OH; D_2_O exchangeable); ^13^C-NMR spectrum (DMSO, δ ppm): 14.73, 22.63, 25.16, 34.47, 45.85, 61.21, 79.77, 103.99, 114.11, 116.72, 124.64, 126.69, 129.55, 130.33, 132.17, 135.60, 136.38, 142.31, 143.18, 147.61, 161.00, 172.53. MS, *m/z* (%): 447 (M^+^-87, 100), 449 ([M^+^ + 2]-87, 32). Analysis calc. for C_28_H_27_ClN_4_O_3_S (534.15):C, 62.85; H, 5.09; Cl, 6.63; N, 10.47; S, 5.99. Found: C, 62.56; H, 4.80; Cl, 6.34; N, 10.18; S, 5.71.

### 3.3. Anticancer Activity

Carried out at Al-Azhar University, The Regional Center for Mycology and Biotechnology, Cairo, Egypt.

#### 3.3.1. Chemicals

Dimethyl sulfoxide (DMSO), crystal violet, and trypan blue dye were purchased from Sigma (St. Louis, MO, USA). Fetal Bovine serum, DMEM, RPMI-1640, HEPES buffer solution, L-glutamine, gentamycin, and 0.25% Trypsin-EDTA were purchased from Lonza (Basel, Switzerland). Crystal violet stain (1%): It was composed of 0.5% (*w*/*v*) crystal violet and 50% methanol then made up to volume with ddH_2_O and filtered through a Whatmann No.1 filter paper purchased from Sigma-Aldrich, Saint Louis, MO, USA.

#### 3.3.2. Mammalian Cell Lines

MCF-7 (human breast cancer cell line) and HepG-2 (human liver cancer cell line) were obtained from the American Type Culture Collection (Rockville, MD, USA)

#### 3.3.3. Cell Line Propagation

The cells were propagated in Dulbecco’s modified Eagle’s medium (DMEM) supplemented with 10% heat-inactivated fetal bovine serum, 1% L-glutamine, HEPES buffer, and 50 µg/mL gentamycin. All cells were maintained at 37 °C in a humidified atmosphere with 5% CO_2_ and were subcultured two times a week. Cytotoxicity evaluation using viability assay: For cytotoxicity assay, the cells were seeded in a 96-well plate at a cell concentration of 1 × 10^4^ cells per well in 100 µL of growth medium. Fresh medium containing different concentrations of the test sample was added after 24 h of seeding. Serial two-fold dilutions of the tested chemical compound were added to confluent cell monolayers dispensed into 96-well, flat-bottomed microtiter plates (Falcon, NJ, USA) using a multichannel pipette. The microtiter plates were incubated at 37 °C in a humidified incubator with 5% CO_2_ for a period of 48 h. Three wells were used for each concentration of the test sample. Control cells were incubated without a test sample and with or without DMSO. The little percentage of DMSO present in the wells (maximal 0.1%) was found not to affect the experiment. After incubation of the cells for at 37 °C, various concentrations of the sample were added, and the incubation was continued for 24 h and viable cells yield was determined through a colorimetric method [79].

In brief, after the end of the incubation period, media were aspirated, and the crystal violet solution (1%) was added to each well for at least 30 minutes. The stain was removed, and the plates were rinsed using tap water until all excess stain was removed. Glacial acetic acid (30%) was then added to all wells and mixed thoroughly, and then the absorbance of the plates was measured after they were gently shaken on Microplate reader (TECAN, Inc.), using a test wavelength of 490 nm. All results were corrected for background absorbance detected in wells without added stain. Treated samples were compared with the cell control in the absence of the tested compounds. All experiments were carried out in triplicate. The cell cytotoxic effect of each tested compound was calculated. The optical density was measured with the microplate reader (SunRise, TECAN, Inc, Zanker Road, San Jose, CA, USA) to determine the number of viable cells, and the percentage of viability was calculated as [1 − (ODt/ODc)] × 100%, where ODt is the mean optical density of wells treated with the tested sample and ODc is the mean optical density of untreated cells. The relation between surviving cells and drug concentration was plotted to get the survival curve of each tumor cell line after treatment with the specified compound.

IC_50_ calculations. To assess the compounds’ anticancer potency, the IC_50_ values (the concentration that inhibited cell viability to 50% of the control) were determined. The IC_50_ values were calculated from the best-fit (R^2^ > 0.95) of the Hill slope curve to experimental data using nonlinear regression analysis in Graph Pad Prism (Graph Pad Software Version 7, San Diego, CA. USA), according to the formula:

Y = 100/1 +10^((LogIC50 − X)^^× HillSlope)^,

where X = log of dose, Y = growth inhibition value normalized to control, and HillSlope = unitless slope factor or Hill slope [80].

### 3.4. In Vitro Antidiabetic Assay

Carried out at Al-Azhar University, The Regional Center for Mycology and Biotechnology, Cairo, Egypt.

#### 3.4.1. Alpha-Amylase Inhibitory Activity

##### Chemicals

Alpha-amylase and 3,5-dinitro salicylic acid (DNS) were purchased from Sigma-Aldrich, Saint Louis, MO, USA, while starch, sodium dihydrogen phosphate, and di-sodium hydrogen phosphate were purchased from Hi-Media, PA, USA.

##### Alpha-Amylase Inhibition Method

In the alpha–amylase inhibition method, the enzyme solution was prepared by dissolving α–amylase in 20 mM phosphate buffer (6.9) at a concentration of 0.5 mg/mL. One milliliter of the extract of various concentrations (1000–7.81 μg/mL) and 1 mL of enzyme solution were mixed together and incubated at 25 °C for 10 min. After incubation, 1 mL of starch (0.5%) solution was added to the mixture and further incubated at 25 °C for 10 min. The reaction was then stopped by adding 2 mL of 3,5-dinitro salicylic acid (DNS, color reagent), heating the reaction mixture in a boiling water bath (5 min). After cooling, the absorbance was measured colorimetrically at 565 nm [81]. The inhibition percentage was calculated using the given formula:

Inhibitory activity (%) = (1−As/Ac) × 100,

where As is the absorbance in the presence of test substance and Ac is the absorbance of the control. Acarbose was used as a standard drug. The IC_50_ value was defined as the concentration of alpha–amylase inhibitor to inhibit 50% of its activity under the assay conditions.

#### 3.4.2. Alpha-Glucoidase Inhibitory Activity

##### Chemicals

Alpha-glucosidase (*Saccharomyces cerevisiae*) and 3,5-dinitro salicylic acid (DNS) were purchased from Sigma-Aldrich, Saint Louis, Missouri, USA, while *P*-nitrophenyl-α–*D*-glucopyranoside (*p*-NPG), sodium carbonate (Na_2_CO_3_), sodium dihydrogen phosphate, and di-sodium hydrogen phosphate were purchased from Hi-Media, Pennsylvania, USA.

##### Alpha-Glucoidase Inhibition Method

The alpha–glucosidase inhibitory activity of *B. vulgaris* subspecies *cicla* L. var. *flavescens* leaves different extracts and fractions was carried out according to the standard method with minor modification [82]. In a 96-well plate, reaction mixture containing 50 μL phosphate buffer (100 mM, pH = 6.8), 10 μL alpha–glucosidase (1 U/mL), and 20 μL of varying concentrations of extracts and fractions (1000 to 7.81 μg/mL) was preincubated at 37 ° C for 15 min. Then, 20 μL *P*-NPG (5 mM) was added as a substrate and incubated further at 37 °C for 20 min. The reaction was stopped by adding 50 μL Na_2_CO_3_ (0.1 M). The absorbance of the released *p*-nitrophenol was measured at 405 nm using Multiplate Reader. Acarbose at various concentrations (1000 to 7.81 μg/mL) was included as a standard. Without a test, the substance was set up in parallel as a control, and each experiment was performed in triplicates. The results were expressed as percentage inhibition, which was calculated using the formula:

Inhibitory activity (%) = (1 – As/Ac) × 100,

where As is the absorbance in the presence of test substance and Ac is the absorbance of control. The IC_50_ value was defined as the concentration of alpha-glucosidase inhibitor to inhibit 50% of its activity under the assay conditions.

## 4. Conclusion

Our study concerned the preparation of new spirothiazolidene derivatives and its fused analogs, which were prepared and elucidated using spectral and elemental analysis. Generally, the type of heterocyclic framework of these derivatives had a notable effect on the anticancer and antidiabetic activity. The prepared compounds were screened for anticancer activity towards the breast and liver human cell line. Compounds **6**, **14**, and **16**, containing spirothiazolopyridine–carbonitrile derivatives with amino or acetic acid or propanoic acid groups showed excellent anticancer activity at all concentrations. Furthermore, it is apparent that compounds **6** and **9** containing amino spirothiazolopyridine–carbonitrile and pyrazolo spirothiazolidine groups display high activity against both of alpha-amylase and alpha-glucosidase enzyme in all concentrations.

## Figures and Tables

**Table 1:** Effect of treatment at various concentrations of prepared compounds on MCF-7 and HepG-2 cell cytotoxicity.

Compounds	Cell Lines		Concentration in (µg/mL)									IC_50_ (µg/mL)
			0	3.90	7.8	15.6	31.25	62.5	125	250	500	
**Doxorubicin**	MCF-7	% of cell viability	100	24.98	19.89	15.46	6.93	5.07	3.21	2.36	1.51	0.35
	HepG-2	% of cell viability	100	25.59	20.81	18.13	13.05	6.13	4.22	2.7	1.72	0.36
**4**	MCF-7	% of cell viability	100	100	100	100	98.03	90.66	78.27	47.96	31.62	242
	HepG-2	% of cell viability	100	92.22	77.22	56.55	42.1	31.19	28.55	18.36	8.96	15.87
**6**	MCF-7	% of cell viability	100	88.23	74.95	63.37	48.92	34.25	29.38	21.64	13.87	30
	HepG-2	% of cell viability	100	80.41	63.57	47.28	39.56	30.69	21.97	14.36	7.96	14.3
**8**	MCF-7	% of cell viability	100	100	99.12	94.57	86.72	78.64	63.96	39.34	28.15	196
	HepG-2	% of cell viability	100	89.87	64.11	48.11	39.87	29.88	22.65	17.65	7.58	16.85
**12**	MCF-7	% of cell viability	100	100	98.49	90.12	82.89	66.25	54.82	35.76	26.57	157
	HepG-2	% of cell viability	100	92.1	88.14	69.01	40	30	20.09	14.33	8.66	11.44
**14**	MCF-7	% of cell viability	100	68.94	54.28	47.31	39.22	34.18	26.43	18.92	9.38	12.6
	HepG-2	% of cell viability	100	78.63	52.19	40.76	31.78	24.95	18.56	11.79	6.42	9.29
**16**	MCF-7	% of cell viability	100	97.37	95.24	89.13	75.42	61.35	37.53	29.48	18.62	92.3
	HepG-2	% of cell viability	100	96.41	89.06	78.59	62.37	41.7	32.94	20.41	13.28	50

**Table 2:** Effect of treatment at various concentrations of prepared compounds on alpha-amylase inhibitor and alpha-glucosidase inhibitor.

Compounds	* Mean of Inhibitory%	Concentration in (µg/mL)									** IC_50_ (µg/mL)
		0	7.81	15.63	31.25	62.5	125	250	500	1000	
**Acarbose **	α–amylase ± SD	0	37.81 ± 1.2	40.75 ± 1.5	48.84 ± 1.2	59.31 ± 1.5	60.17 ± 0.63	69.37 ± 1.2	80.14 ± 0.58	86.32 ± 0.63	34.71
	α–glucosidase ± SD	0	32.15 ± 0.58	43.28 ± 1.2	50.31 ± 1.5	60.14 ± 0.72	63.42 ± 2.1	71.34 ± 1.5	86.34 ± 1.2	90.10 ± 0.58	30.57
**1**	α–amylase ± SD	0	0	7.35 ± 0.35	12.35 ± 1.2	24.35 ± 0.58	38.31 ± 0.63	48.98 ± 2.1	54.31 ± 1.2	58.34 ±1.5	297.8
	α–glucosidase ± SD	0	0	5.34 ± 0	16.25 ± 0	38.14 ± 1.5	50.98 ± 0.58	63.15 ± 1.5	69.32 ± 1.5	71.34 ± 1.3	120.22
**2**	α–amylase ± SD	0	0	0	10.36 ± 1.5	23.22 ± 0.63	29.34 ± 1.2	46.35 ± 0.58	53.14 ± 3.1	58.32 ± 1.5	421.42
	α–glucosidase ±SD	0	0	0	18.37 ± 2.1	23.15 ± 1.5	31.25 ± 0.63	44.38 ± 0.58	56.74 ± 1.5	61.38 ± 1.2	363.67
**6**	α–amylase ± SD	0	0	14.63 ± 1.2	22.38 ± 2.5	43.12 ± 1.5	56.39 ± 2.1	61.35 ± 1.2	69.28 ± 0.63	72.51 ± 1.5	94.9
	α–glucosidase ± SD	0	0	11.32 ± 1.2	31.54 ± 1.5	49.75 ± 0.72	64.35 ± 0.58	71.21 ± 3.1	73.42 ± 1.5	80.35 ± 0.63	63.6
**7**	α–amylase ± SD	0	0	6.85 ± 1.2	18.14 ± 1.5	31.75 ± 2.5	42.93 ± 3.1	50.38 ± 1.5	59.32 ± 0.58	63.25 ± 1.2	239.37
	α–glucosidase ± SD	0	15.37 ± 0.72	23.41 ± 2.1	41.57 ± 1.5	49.21 ± 1.5	50.11 ± 2.1	61.58 ± 0.58	72.46 ± 1.2	79.21 ± 0.63	117.36
**8**	α–amylase ± SD	0	7.39 ± 1.2	19.37 ± 0.58	25.13 ± 0.63	37.21 ± 1.5	46.95 ± 2.1	52.17 ± 2.1	60.24 ± 0.58	66.32 ± 1.5	199.01
	α–glucosidase ± SD	0	24.31 ± 3.1	30.24 ± 1.2	42.16 ± 1.5	49.31 ± 0.58	50.14 ± 0.63	59.42 ± 0.72	62.13 ± 2.1	74.35 ± 1.5	114.45
**9**	α–amylase ± SD	0	12.78 ± 2.1	20.17 ± 1.2	31.22 ± 0.63	52.48 ± 0.72	58.42 ± 1.5	61.85 ± 1.2	69.38 ± 0.58	78.35 ± 0.63	58.85
	α–glucosidase ± SD	0	6.34 ± 0	22.34 ± 2.1	36.58 ± 1.5	56.34 ± 0.58	65.32 ± 0.63	70.14 ± 1.2	74.35 ± 2.5	81.32 ± 1.5	52.47
**13**	α–amylase ± SD	0	0	10.35 ± 1.2	14.32 ± 1.5	29.35 ± 0.58	41.33 ± 0.72	49.31 ± 0.72	57.32 ± 1.2	60.30 ± 1.5	271.3
	α–glucosidase ± SD	0	0	7.84 ± 2.1	18.38 ± 1.2	39.34 ± 0.58	51.32 ± 0.86	64.35 ± 2.1	70.41 ± 1.2	72.51 ± 0.63	118.11
**16**	α–amylase ± SD	0	0	0	0	0	7.63 ± 1.5	24.84 ± 1.2	32.45 ± 0.58	41.31 ± 0.63	>1000
	α–glucosidase ±SD	0	0	0	11.32 ± 1.5	15.31 ± 0.58	21.34 ± 1.5	34.21 ± 0.63	48.32 ± 0.58	60.24 ± 1.2	570.4

* All determinations were carried out in a triplicate manner, and values are expressed as the mean ± SD. ** The IC_50_ value is defined as the concentration of inhibitor to inhibit 50% of its activity under the assayed conditions.

## Supplementary Materials

The following are available online at https://www.mdpi.com/1420-3049/24/13/2511/s1, evaluation of cytotoxicity against HepG-2 and MCF-7cell lines.
