# Supplementary material for: Development of a Novel Series of Anticancer and Antidiabetic: Spirothiazolidines Analogs
Source: Molecules. 2019 Jul 9;24(13):2511. doi: 10.3390/molecules24132511 (PMC6650905; doi:10.3390/molecules24132511)
Supplement: Supplementary file 1 [file molecules-24-02511-s001.pdf]

Supplementary Material

Development of a Novel Series of Anticancer and Antidiabetic: Spirothiazolidines Analogs

Eman M. Flefel [1,2] , Walaa I. El-Sofany [1], Reem A.K. Al-Harbi [2] and Mahmoud El-Shahat [1*]

[1] Department of Photochemistry, Chemical Industries Research Division, National Research Centre, 33 EL-Bohouth St., Dokki 12622, Giza, Egypt; emanmflefel@yahoo.com (E.M.F.); walaa.elsofany@gmail.com (W.I.E.-S)

[2] Department of Chemistry, College of Science, Taibah University, Al-Madinah Al-Monawarah 1343, Saudi Arabia; reemal94@hotmail.com

* Correspondence: mahmoudelshahat@gmail.com; Tel.: +202-01112234243

Received: 13 June 2019; Accepted: 03 July 2019; Published: date

Evaluation of cytotoxicity against HepG-2 cell line

Requester Data:

Name: **Dr. Walaa Ibrahim**
Authority: **National Research Center**
Sample Code: **6**

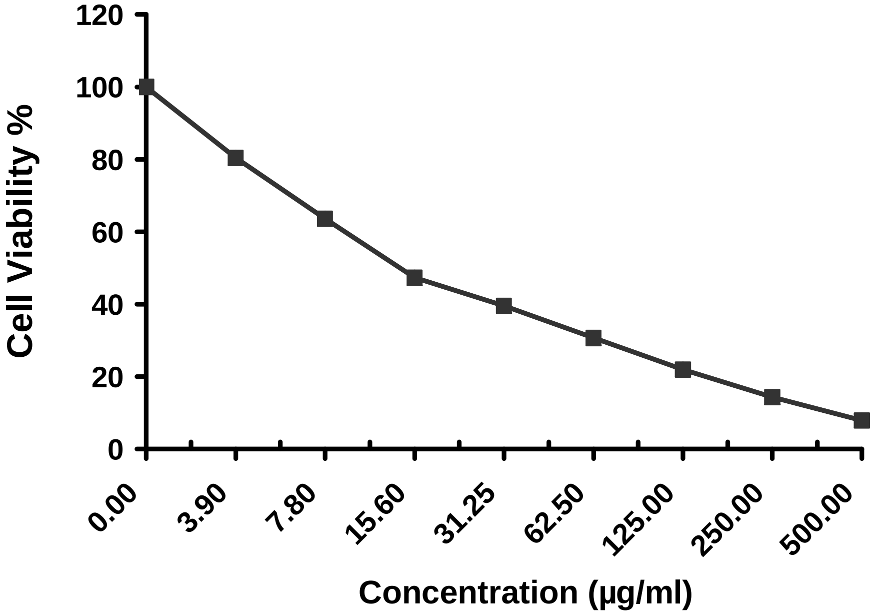

Sample conc. (µg/ml)	Viability %
500	7.95
250	14.36
125	21.97
62.5	30.69
31.25	39.56
15.6	47.28
7.8	63.57
3.9	80.41
0	100

Comment:

Inhibitory activity against Hepatocellular carcinoma cells was detected under these experimental conditions with IC_{50} = 14.3 µg/ml.

Sample Code: 14

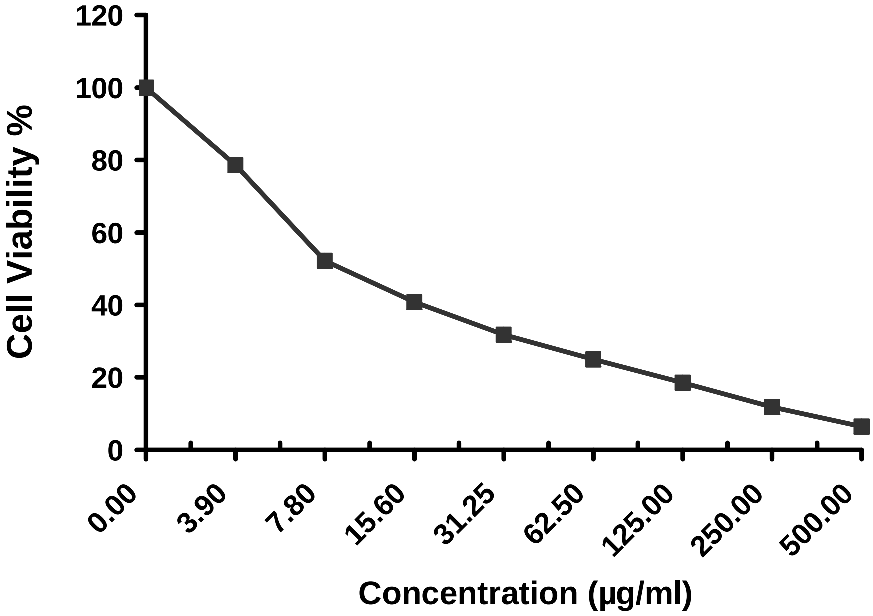

Sample conc. (µg/ml)	Viability %
500	6.42
250	11.79
125	18.56
62.5	24.95
31.25	31.78
15.6	40.76
7.8	52.19
3.9	78.63
0	100

Comment:

Inhibitory activity against Hepatocellular carcinoma cells was detected under these experimental conditions with IC_{50} = 9.29 µg/ml.

Sample Code: 16

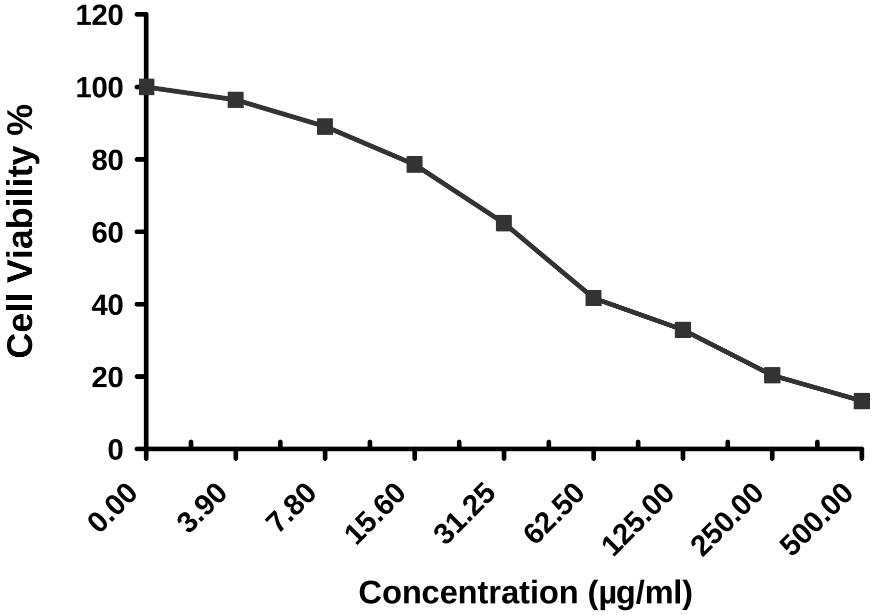

Sample conc. (µg/ml)	Viability %
500	13.28
250	20.41
125	32.94
62.5	41.70
31.25	62.37
15.6	78.59
7.8	89.06
3.9	96.41
0	100

Comment:

Inhibitory activity against Hepatocellular carcinoma cells was detected under these experimental conditions with IC_{50} = 50 µg/ml.

Sample Code: (Doxorubicin Reference Standard)

HepG-2 Doxorubicin

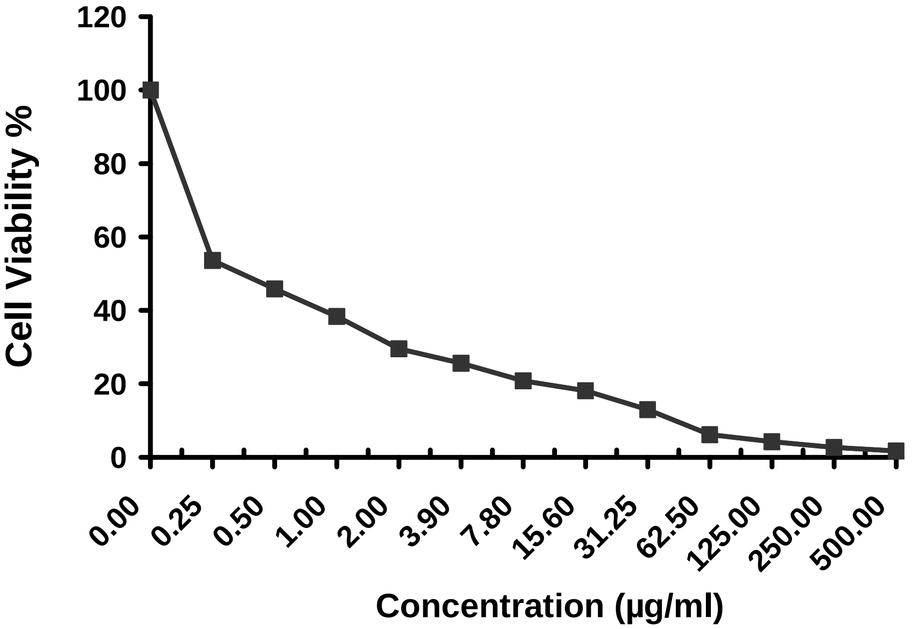

Sample conc. (µg/ml)	Viability %	Inhibition %	S.D. (±)
500	1.72	98.28	0.42
250	2.70	97.30	0.50
125	4.22	95.78	0.36
62.5	6.13	93.87	0.39
31.25	13.05	86.95	0.72
15.6	18.13	81.87	1.16
7.8	20.81	79.19	1.22
3.9	25.59	74.41	0.89
2	29.50	70.50	0.75
1	38.39	61.61	1.05
0.5	45.84	54.16	0.67
0.25	53.57	46.43	0.72
0	100	0.00	

***Comment*:**

Inhibitory activity against Hepatocellular carcinoma cells was detected under these experimental conditions with IC_{50} = 0.36 µg/ml.

<u>**Evaluation of cytotoxicity against MCF-7 cell line**</u>

Requester Data:

Name: **Dr. Walaa Ibraheem Ahmed**
Authority: **National Research Center**
Sample Code: **6**

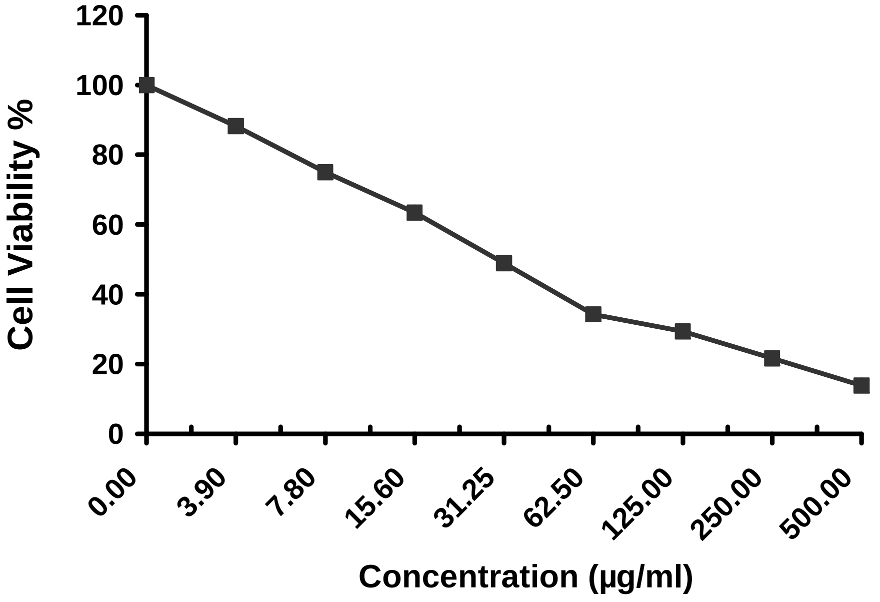

Sample conc. (µg/ml)	Viability %
500	13.87
250	21.64
125	29.38
62.5	34.25
31.25	48.92
15.6	63.37
7.8	74.95
3.9	88.23
0	100

Comment:

Inhibitory activity against Breast carcinoma cells was detected under these experimental conditions with IC_{50} = 30 µg/ml.

Sample Code: 8

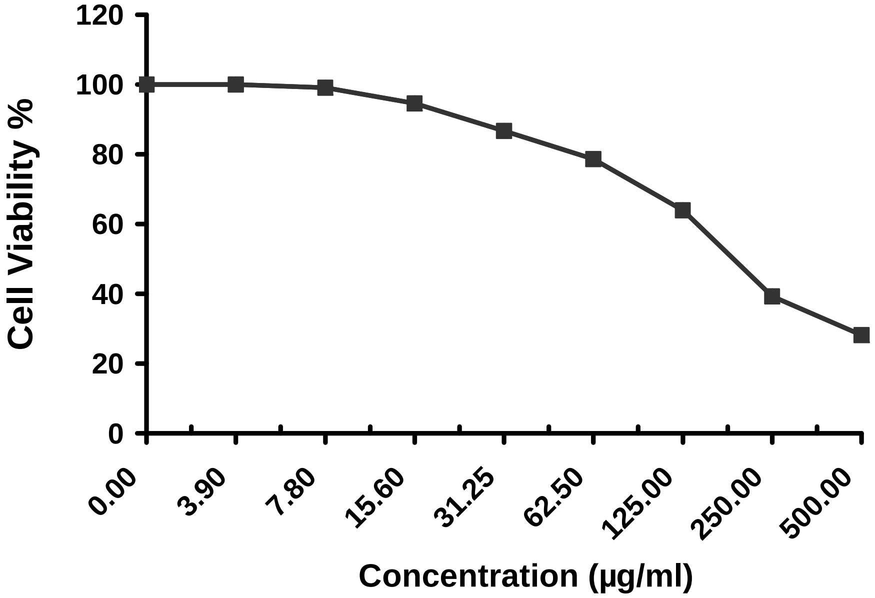

Sample conc. (µg/ml)	Viability %
500	28.15
250	39.34
125	63.96
62.5	78.64
31.25	86.72
15.6	94.57
7.8	99.12
3.9	100
0	100

Comment:

Inhibitory activity against Breast carcinoma cells was detected under these experimental conditions with IC_{50} *= 196 µg/ml.*

Sample Code: 12

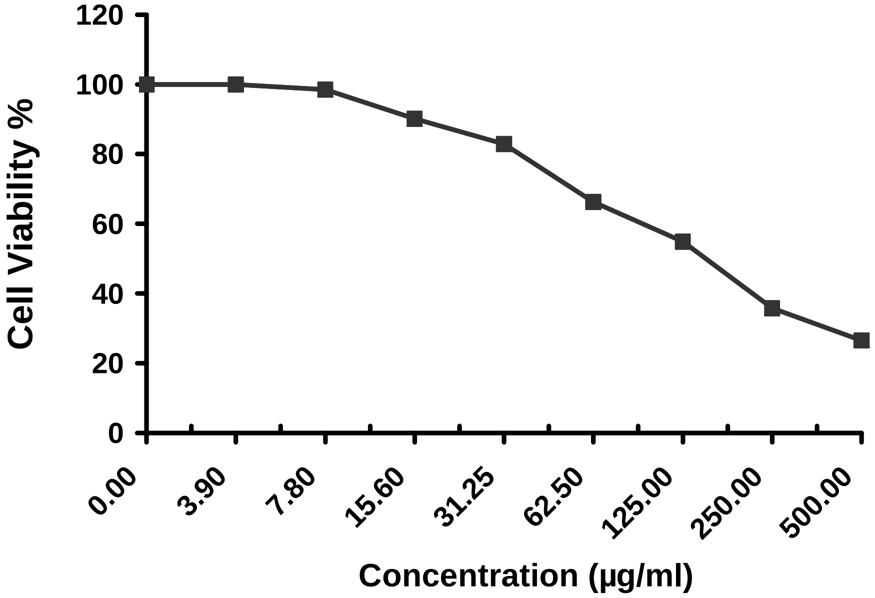

Sample conc. (µg/ml)	Viability %
500	26.57
250	35.76
125	54.82
62.5	66.25
31.25	82.89
15.6	90.12
7.8	98.49
3.9	100
0	100

***Comment*:**

Inhibitory activity against Breast carcinoma cells was detected under these experimental conditions with IC_{50} = 157 µg/ml.

Sample Code: 14

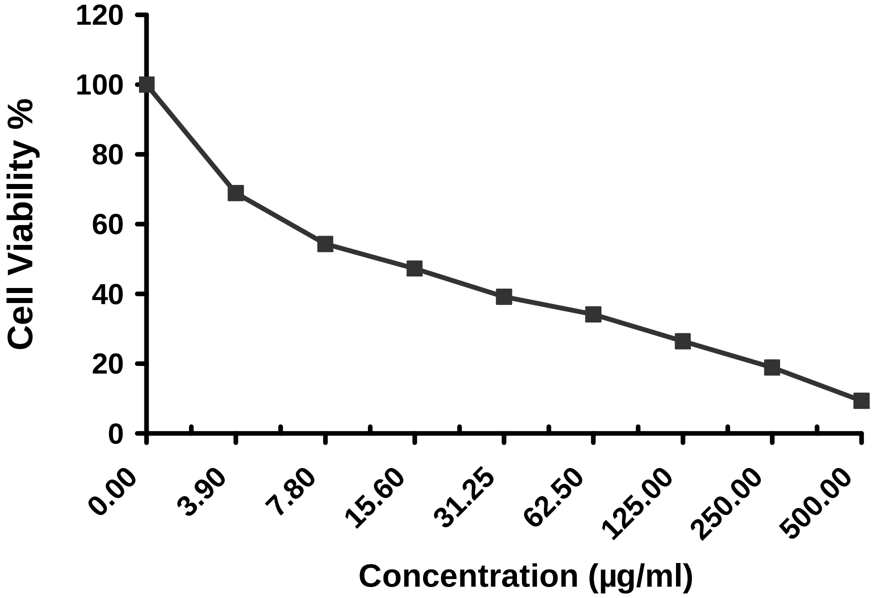

Sample conc. (μg/ml)	Viability %
500	9.38
250	18.92
125	26.43
62.5	34.18
31.25	39.22
15.6	47.31
7.8	54.28
3.9	68.94
0	100

***Comment*:**

Inhibitory activity against Breast carcinoma cells was detected under these experimental conditions with IC_{50} = 12.6 μg/ml.

Sample Code: 16

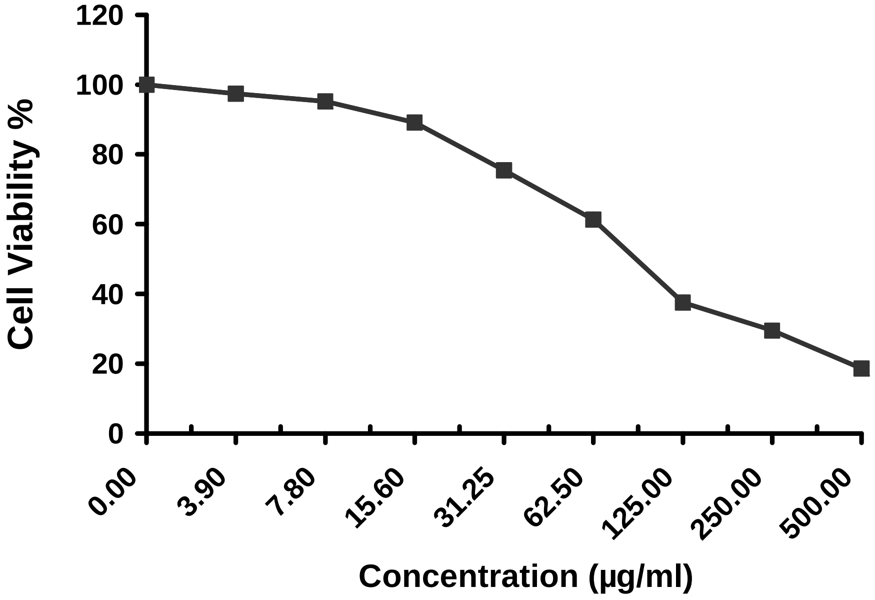

Sample conc. (µg/ml)	Viability %
500	18.62
250	29.48
125	37.53
62.5	61.35
31.25	75.42
15.6	89.13
7.8	95.24
3.9	97.37
0	100

***Comment*:**

Inhibitory activity against Breast carcinoma cells was detected under these experimental conditions with IC_{50} = 92.3 µg/ml.

Sample Code: 4

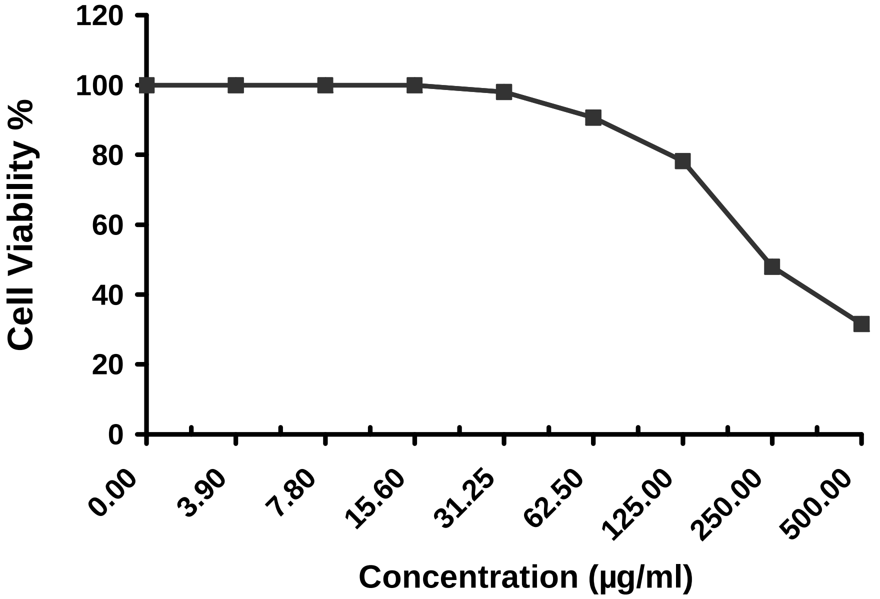

Sample conc. (µg/ml)	Viability %
500	31.62
250	47.96
125	78.27
62.5	90.66
31.25	98.03
15.6	100
7.8	100
3.9	100
0	100

***Comment*:**

Inhibitory activity against Breast carcinoma cells was detected under these experimental conditions with IC_{50} = 242 µg/ml.

Sample Code: (Doxorubicin Reference Standard)

MCF-7 **Doxorubicin**

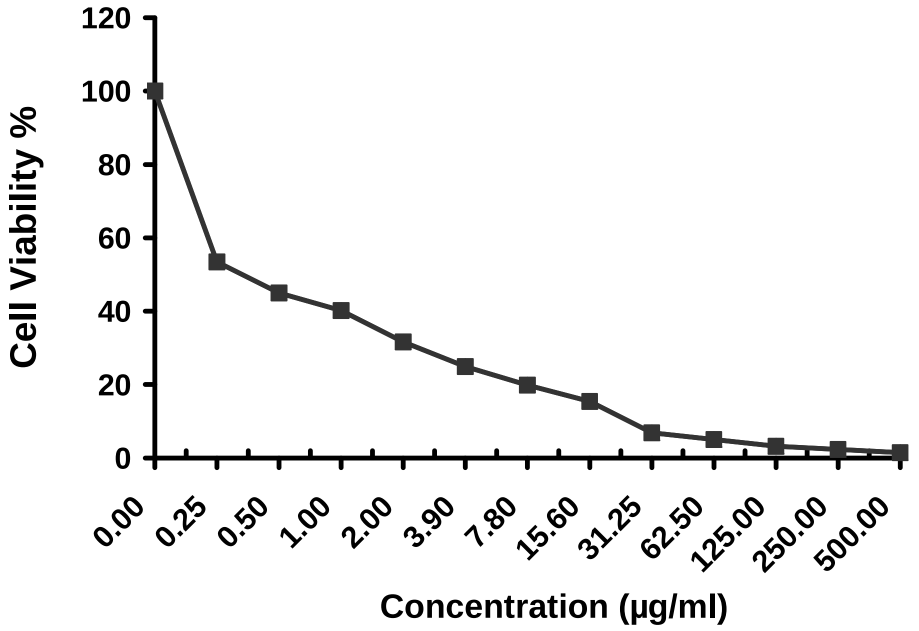

Sample conc. (µg/ml)	Viability %	Inhibition %	S.D. (±)
500	1.51	98.49	0.17
250	2.36	97.64	0.26
125	3.21	96.79	0.21
62.5	5.07	94.93	0.32
31.25	6.93	93.07	0.29
15.6	15.46	84.54	1.07
7.8	19.89	80.11	1.27
3.9	24.98	75.02	1.30
2	31.69	68.31	0.82
1	40.17	59.83	1.53
0.5	45.02	54.98	1.11
0.25	53.41	46.59	0.85
0	100	0.00	

Comment:

Inhibitory activity against Breast carcinoma cells was detected under these experimental conditions with IC_{50} = 0.35 µg/ml.
